# Characteristics associated with antenatally unidentified small-for-gestational-age fetuses: prospective cohort study nested within the DESiGN randomized control trial

**DOI:** 10.1002/uog.26091

**Published:** 2023-03-01

**Authors:** S. Relph, M. C. Vieira, A. Copas, A. Alagna, L. Page, C. Winsloe, A. Shennan, A. Briley, M. Johnson, C. Lees, D. A. Lawlor, J. Sandall, A. Khalil, D. Pasupathy, on behalf of the DESiGN Trial Team and DESiGN Collaborative Group

**Affiliations:** 1 Department of Women and Children’s Health, School of Life Course Sciences, Faculty of Life Sciences and Medicine, King’s College London, London, UK; 2 Department of Obstetrics and Gynaecology, School of Medical Sciences, University of Campinas (UNICAMP), Campinas, SP, Brazil; 3 Centre for Pragmatic Global Health Trials, Institute for Global Health, University College London, London, UK; 4 Guy’s & St Thomas’ Charity, London, UK; 5 West Middlesex University Hospital, Chelsea & Westminster Hospital NHS Foundation Trust, Isleworth, UK; 6 Caring Futures Institute, Flinders University and North Adelaide Local Health Network, Adelaide, Australia; 7 Department of Surgery and Cancer, Imperial College London, London, UK; 8 Department of Metabolism, Digestion and Reproduction, Imperial College London, London, UK; 9 Population Health Science, Bristol Medical School, University of Bristol, Bristol, UK; 10 Bristol NIHR Biomedical Research Centre, Bristol, UK; 11 MRC Integrative Epidemiology Unit, University of Bristol, Bristol, UK; 12 Fetal Medicine Unit, St George’s University Hospitals NHS Foundation Trust, London, UK; 13 Molecular & Clinical Sciences Research Institute, St George’s University of London, London, UK; 14 Reproduction and Perinatal Centre, Faculty of Medicine and Health, University of Sydney, NSW, Australia

**Keywords:** Small-for-gestational age, fetal growth, antenatal screening, ultrasound patterns, risk factors, estimated fetal weight, maternal characteristics

## Abstract

**Objective:**

To identify the clinical characteristics and patterns of ultrasound use amongst pregnancies with antenatally unidentified SGA, compared with those in which it is identified, to understand how to better design interventions that improve antenatal SGA identification.

**Methods:**

This was a prospective cohort study of singleton, non-anomalous, small-for-gestational age (SGA, birth weight<10th centile) neonates born after 24+0 gestational weeks at 13 UK sites, recruited for the baseline period and control arm of the DESiGN trial. Pregnancy with antenatally unidentified SGA was defined if there was no scan or if the final scan showed estimated fetal weight (EFW) at 10^th^ centile or above. Identified SGA was defined if EFW was below 10th centile at the last scan. Maternal and fetal sociodemographic and clinical characteristics were studied for associations with unidentified SGA using unadjusted and adjusted logistic regression models. Ultrasound parameters (gestational age at first growth scan, ultrasound number and frequency) were described, stratified by presence of indications for serial ultrasound. Associations of missed SGA with absolute centile and percentage birthweight difference between the last scan and the birth were also studied by unadjusted and adjusted logistic regression, stratified by duration between the last scan and the birth. but

**Results:**

Of the 15,784 SGA babies included, SGA was not identified antenatally in 78.7% of cases. Of pregnancies with unidentified SGA, 47.1% had no recorded growth scan. Amongst 9,410 pregnancies with complete data on key maternal comorbidities and antenatal complications, the risk of unidentified SGA was lower for women with any indication for serial scans (aOR 0.56, 95% CI, 0.49–0.64), Asian compared with white race (aOR 0.80, 95% CI, 0.69–0.93) and non-cephalic presentation at birth (aOR 0.58, 95% CI, 0.46–0.73). The risk of unidentified SGA was highest among women with BMI of 25.0–29.9 kg/m^2^ (aOR 1.15 (95% CI, 1.01–1.32)) and lowest in those with underweight BMI (aOR 0.61, 95% CI, 0.48–0.76) compared to women with BMI of 18.5–24.9 kg/m^2^. Compared to women with identified SGA, those with unidentified SGA had fetuses of higher SGA birth-weight centile (adjusted mean difference 1.21, 95% CI, 1.18–1.23). Duration between the last scan and birth increased with advancing gestation in pregnancies with unidentified SGA. SGA babies born within a week of the last growth scan had a mean difference between EFW and birth-weight centiles of 19.5 (SD: 13.8) centiles for the unidentified SGA group and 0.2 (SD: 3.3) centiles for the identified SGA group (adjusted mean difference between groups: 19.0 (95% CI: 17.8–20.1) centiles).

**Conclusions:**

Unidentified SGA was more common amongst women without an indication for serial ultrasound, cephalic presentation at birth, BMI 25.0–29.9 kg/m^2^ and less severe SGA. Ultrasound EFW was overestimated in women with unidentified SGA. This demonstrates the importance of improving the accuracy of SGA screening strategies in low-risk populations and continuing performance of ultrasound scans for term pregnancies.

## Introduction

The reduction of stillbirth and perinatal death rates is an international priority.^1^ Between 30% and 50% of stillborn babies are small-for-gestational age (SGA, birth weight <10^th^ centile for gestational age),^2–7^ and being SGA increases the risk of stillbirth 4-fold.^8^ It is therefore accepted that improvements in antenatal detection of SGA fetuses and subsequent perinatal care are needed to reduce the rate of stillbirth.^9^

Current strategies to screen for SGA (or fetal growth restriction (FGR)) during pregnancy involve fundal height measurement and targeted ultrasound for women at low-risk of SGA/FGR and serial fetal ultrasound assessment for women with risk factors for SGA/FGR.^10^ This strategy is associated with a rate of detection of SGA < 50%.^11–18^ Alternatively, universal serial ultrasound screening detects a higher proportion of SGA in research settings, but without replication in routine care.^12, 13^

Improving the rate of antenatal detection of SGA without consequential increase in false-positive diagnoses requires an understanding of maternal and perinatal characteristics of pregnancies in which SGA is not currently identified antenatally. Previous studies have found that FGR was more likely to be detected amongst multiparous women (particularly those with a previous FGR baby), women with lower BMI, those who had assisted conception^19^ and if a third-trimester fetal growth scan had been conducted.^20^ FGR was less likely to be detected if the fetal growth scan was falsely reassuring (EFW or AC>10^th^ centile) and in women cared for in low-risk midwifery-led settings.^20^ However, clinical characteristics included in either study were limited.

This study aimed to identify the clinical characteristics and patterns of ultrasound use amongst pregnancies in which SGA is not identified antenatally, compared with those in which it is identified, to understand how we can better design interventions to improve detection.

## Methods

### Study design

This was a prospective cohort study conducted using data on pregnancies and births collected for the DESiGN trial. DESiGN was a UK randomized cluster controlled trial conducted between 5 November 2016 and 28 March 2019, which compared the clinical effectiveness of the Growth Assessment Protocol (GAP) in the antenatal detection of SGA with that of standard care, finding no difference in primary outcome between the strategies and a weak economic case for replacing standard care with GAP.^21, 22^ Detailed descriptions of the trial and data collection methods have been published previously.^21, 23, 24^

For this analysis, we included only those pregnancies in which the neonate had SGA (defined as birth weight below the 10^th^ centile for gestational age on population reference charts^25^) after 24+0 gestational weeks and was not exposed to the intervention. This included all pregnancies from control clusters and any pregnancies in intervention clusters that occurred prior to the implementation of GAP. Multiple pregnancies (i.e. twins) and those with antenatally diagnosed fetal abnormalities were excluded. Women and babies in whom SGA detection status could not be determined because ultrasound data were missing during an entire trial phase at a cluster site (occurring at two clusters) were also excluded. This study has been reported according to the recommendations of the STROBE statement for observational studies.^26^

### Defining antenatally identified and unidentified cases of SGA

Antenatally unidentified SGA was defined as a pregnancy in which the neonate was diagnosed as SGA at birth (birth weight <10^th^ centile on population birth-weight charts^25^) but for which there was no evidence that an antenatal ultrasound diagnosis had been made i.e. the woman did not undergo growth scans or EFW at the last fetal growth scan (defined as any scan with fetal biometry conducted after 24^+0^ weeks’) was above the 10^th^ centile for gestational age. Identified SGA was defined as a pregnancy in which an antenatal diagnosis of SGA had been made correctly i.e. EFW at the last fetal growth ultrasound was below the 10^th^ centile for gestational age. This outcome was chosen because clinical guidelines on the management of pregnancies with suspected SGA currently commonly apply the EFW<10^th^ centile threshold, and decisions regarding timing and mode of birth are largely driven by EFW at the last scan. EFW was assessed using Hadlock fetal growth charts.^27^

### Exposures

The maternal and fetal characteristics studied included maternal age, index of socioeconomic deprivation quintile, race (black, white, Asian, mixed, other), BMI (<18.5 kg/m^2^, 18.5–24.9 kg/m^2^, 25.0–29.9 kg/m^2^, 30.0–34.9 kg/m^2^, 35.0–39.9 kg/m^2^, ≥40.0 kg/m^2^), parity (0, 1, 2, 3, ≥4), smoking, maternal comorbidity (pre-existing hypertension and diabetes), antenatal complication (pre-eclampsia, gestational hypertension, gestational diabetes (GDM)), low pregnancy-associated plasma protein-A (PAPP-A) (<0.300 MoM, 0.300–0.415 MoM, >0.415 MoM), non-cephalic presentation at birth and birth-weight centile (continuous or <3^rd^ centile, 3^rd^–4.9^th^ centile, 5^th^–10^th^ centile). Categories were chosen according to those used in routine clinical practice, including existing risk-stratification models. A composite exposure category was also developed to include any reported risk factor for SGA, indicating need for serial fetal growth scans during pregnancy (age≥40years, BMI≥35 kg/m^2^, smoking, any of the above maternal comorbidities or antenatal complications, PAPP-A≤0.415 MoM). The maternal comorbidities and antenatal complications were included because each raises the risk of SGA and is therefore an indication for serial fetal growth scans in pregnancy, although the list is limited to indications for which data were accessible.

To assess the patterns of ultrasound use when screening for SGA, only fetal growth scans at which EFW was calculated (or could be calculated using recorded biometry) after 24+0 gestational weeks were studied. Scans were categorized into screening and surveillance scans based on when EFW was first identified to be below the 10^th^ centile: all scans before and including the first scan with EFW<10^th^ centile were categorized as screening scans, while all scans after the scan at which EFW was first below the 10^th^ centile were categorized as surveillance scans.

The following characteristics were considered to assess the patterns of ultrasound use: gestational age at the time of first fetal growth scan, frequency of serial screening scans (mean and categorical: 3-week, 4-week, > 4-week intervals), time between the last (screening or surveillance) scan and birth, and difference between EFW at the last scan and birth weight, expressed in terms of absolute centiles (EFW centile minus birthweight centile) and as weight difference presented as a percentage of birth weight (EFW minus birthweight, divided by birthweight). The calculation of mean screening frequency accounted for different gestational ages at the time of commencing serial screening scans (e.g. because of indications that arise later in pregnancy), antenatal diagnosis of SGA that stops the screening period and birth itself by dividing the period from the first scan until the last screening scan by *n* − 1 (where *n* is the number of screening scans performed). For this analysis, only pregnancies that had at least two screening scans could be included.

### Management of missing data

Patterns of missing data were summarized for each characteristic and outcome using descriptive statistics. Missing data were multiply imputed as described previously.^24^ The primary analysis of factors associated with SGA detection status used imputed data on demographics and growth status, but comorbidities, antenatal complications and fetal presentation were not imputed and were therefore analyzed based on availability. If multiple imputation was used, only percentages and not numbers were provided (except to approximate the total number of included births for each analysis), since frequencies are averaged across 10 imputed datasets. Given that PAPP-A is an important characteristic (when low, it is an indication for serial fetal growth ultrasound), but there was wide variation in its availability, missing data on PAPP-A were included as an exposure category, and for analysis where PAPP-A was studies, sites that did not provide data on it were excluded. For the study of ultrasound patterns, it was assumed that pregnancies without a record of a fetal growth scan did not undergo a scan (sensitivity analysis was conducted to test the impact of this assumption and is described below). Rubin’s rules were used for analysis of imputed data.^28^

### Statistical analysis

The number and proportion of pregnancies in which the neonate was SGA at birth and in which this was diagnosed antenatally were calculated. Characteristics of pregnancies in which SGA was not identified were summarized using descriptive statistics (percentage or mean (SD), as appropriate). Characteristics of pregnancies with unidentified SGA were then compared with those of pregnancies in which SGA was identified using unadjusted and adjusted logistic regression, with results presented as odds ratios. Adjustments were made using all other demographic and clinical characteristics (age, index of socioeconomic deprivation quintile, race, BMI, parity and smoking status), allocated birth-weight centile of the neonate, and maternal comorbidities and antenatal complications. Given that the data were collected from a cluster trial population, all models were also adjusted for the cluster site and trial phase to account for clustering and temporal changes.

Patterns of ultrasound use for screening were also summarized using descriptive statistics. The impact of time interval between last scan and birth on SGA detection was assessed on unadjusted and adjusted regression, as described above. However, for this analysis, adjustments were made using trial factors only (cluster site and trial phase). To determine the impact of ultrasound patterns on the rate of detection of SGA amongst women with and those without an indication for serial fetal ultrasound scans, the comparisons were stratified by the presence or absence of an indication; this available-case analysis was conducted amongst women who had complete information on presence or absence of comorbidities and antenatal complications, with antenatal care at sites that provided data on PAPP-A.

### Sensitivity analysis

The analyses were repeated to determine whether any of the methodological choices had influenced the findings. The analysis was first repeated using only observed (i.e. non-imputed) data (5,307 women with complete data on comorbidities and antenatal complications, of which 4,129 (77.8%) had unidentified SGA; larger sample (*n* = 15,247) with complete data at least on SGA status for analysis of ultrasound patterns, with unidentified SGA in 11,897 (78.0%) cases). The second sensitivity analysis used only pregnancies (*n* = 12,122, including 9,164 (75.6%) with unidentified SGA) in which there was evidence of a presumed anomaly scan (scan conducted between 18+0 and 24+0 gestational weeks) to determine the effect of having continuous third-trimester care at the same cluster site and definite evidence of an ultrasound record. This second analysis was conducted to test the assumption that women who had no record of a fetal growth scan at the cluster site at which they gave birth had not undergone one at that site or elsewhere.

## Results

**Figure 1:**
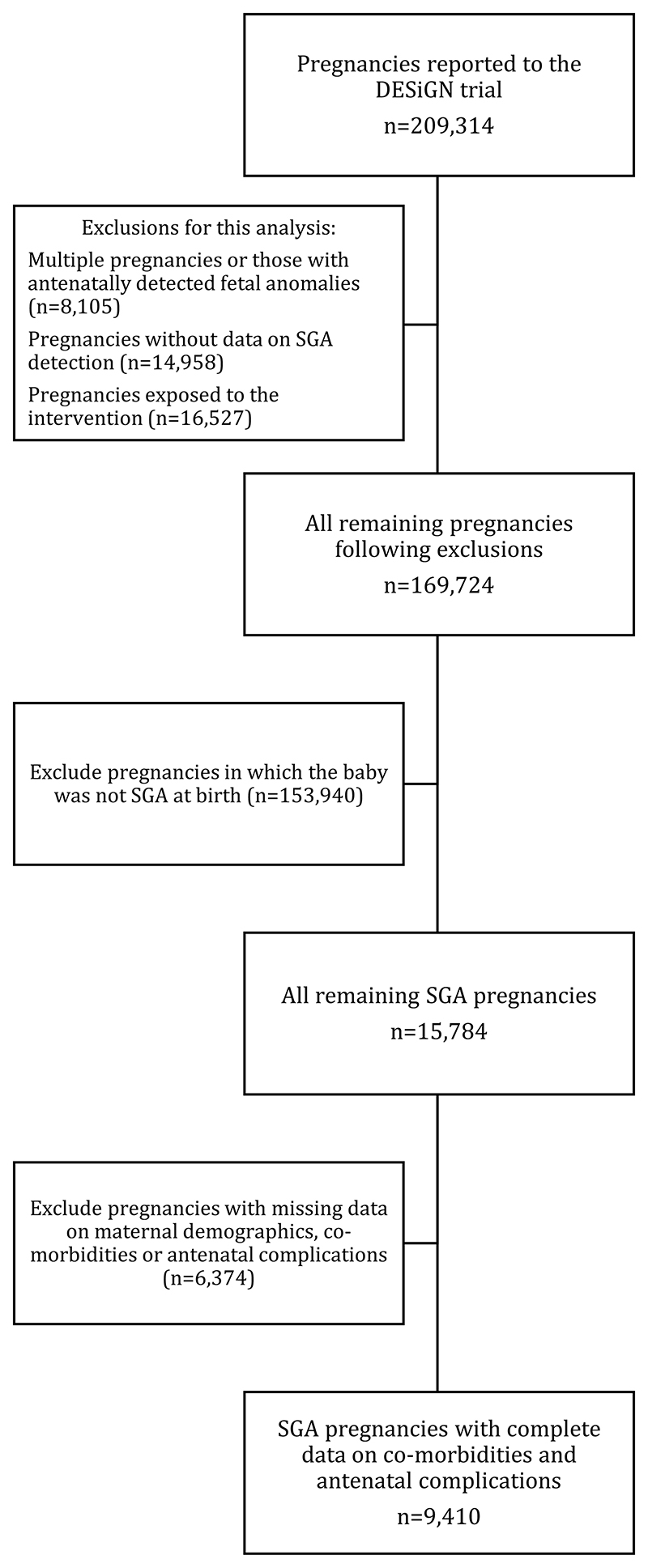
Flowchart summarizing study population of pregnancies (imputed data) in which neonate was diagnosed as small-for-gestational age (SGA) at birth, which were included from the DESiGN (DEtection of Small for GestatioNal age fetus) trial^21^.

Of the 169,724 pregnancies included in the control arm of the DESiGN randomized controlled trial, 9.3% (n≈15,784) were SGA at birth and were included in this study. The characteristics, maternal and neonatal outcomes, and test performance statistics observed in the wider control arm of the trial population (including non-SGA births) during the baseline and outcome periods have been reported elsewhere.^21^ Of these, SGA was not identified antenatally in ≈12,416 (78.7%) cases. Following exclusion of pregnancies with missing data on maternal comorbidities and antenatal complications, ≈9410 pregnancies were available for the assessment of maternal and fetal characteristics associated with unidentified SGA (Figure 1).

### Factors associated with unidentified SGA

Maternal and perinatal characteristics are summarized in according to SGA detection status. Amongst women in whom SGA was unidentified, there was a lower proportion of those aged 40 years or over (3.7% *vs* 5.2%), women with BMI <18.5 kg/m^2^ (5.0% *vs* 7.5%), smokers (8.7% *vs* 10.4%) or cases with any comorbidity (chronic hypertension, 1.9% *vs* 3.0; pre-existing diabetes, 1.2% *vs* 2.1%; pre-eclampsia, 2.6% *vs* 6.9%; gestational hypertension, 1.8% *vs* 3.6%; GDM, 4.6% *vs* 6.8%). Overall, only 31.5% of women with a SGA neonate had any recorded indication for serial fetal growth ultrasound scans (68.5% had no known indication for serial scans); the rate was higher amongst women with identified SGA *vs* those with unidentified SGA (42.8% *vs* 28.5%, *P <* 0.01).

Unadjusted and adjusted comparisons of demographic characteristics and comorbidities or antenatal complications between pregnancies in which SGA was not identified antenatally *vs* those in which it was are presented in Tables 2 and 3. Following mutual adjustment for other factors, the risk of unidentified SGA was lower for women with age over 40 years (aOR, 0.74 (95% CI, 0.56–0.98), p=0.03), women of Asian *vs* white race (aOR, 0.80 (95% CI, 0.69–0.93), global race *P <* 0.01), smokers (aOR, 0.79 (95% CI, 0.66–0.96), p=0.02), those with BMI <18.5 kg/m^2^ *vs* BMI of 18.5–24.9 kg/m^2^ (aOR, 0.61 (95% CI, 0.48–0.76), global BMI p=0.04), those with pre-existing diabetes (aOR, 0.52 (95% CI, 0.34–0.79), *P <* 0.01), gestational diabetes (aOR, 0.64 (95% CI, 0.51–0.80), *P <* 0.01), gestational hypertension (aOR, 0.54 (95% CI, 0.39–0.74), *P <* 0.01), pre-eclampsia (aOR, 0.40 (95% CI, 0.31–0.51), *P <* 0.01), low PAPP-A (aOR, 0.45 (95% CI, 0.32–0.64)for <0.3 MoM and aOR, 0.56 (95% CI, 0.43-0.75) for 0.3-0.415 MoM, P<0.01 for both) or any indication for serial scans (composite aOR, 0.56 (95% CI, 0.49–0.64), *P <* 0.01). Compared to women with BMI of 18.5–24.9 kg/m^2^, risk of missed SGA was significantly higher for women with BMI of 25.0–29.9 kg/m^2^ (aOR, 1.15 (95% CI, 1.01–1.32)) and non-significantly higher for BMI of 30.0–34.9 kg/m^2^ (aOR, 1.12 (95% CI, 0.91–1.38)) (global BMI p=0.04). An association was not observed for higher BMI categories, although these findings are limited by small numbers.

Overall, 9.7% of SGA neonates were born preterm (<37 completed weeks’ gestation). Compared with neonates in whom SGA was identified antenatally, neonates in whom SGA was not identified antenatally were less likely to be born at early term, preterm and extreme preterm gestational age, and therefore were more likely to be born after 39 weeks’ gestation. Of neonates in whom SGA was not identified antenatally, 61.0% were born after their expected due date. Regarding fetal factors, the risk of antenatally unidentified SGA increased with increasing birth-weight centile (within the range of 0–10^th^ centile, aOR increased by 1.21 (95% CI, 1.18–1.23) per one centile increase (*P <* 0.01)) and was lowest for cases with a non-cephalic presentation at birth (aOR, 0.58 (95% CI, 0.46–0.73), *P <* 0.01) (Table 3).

### Comparison of measures of ultrasound utilization

Where only data from the last ultrasound scan were required, patterns of ultrasound use were investigated in the entire study sample of SGA pregnancies (n≈15,784 across imputed datasets). Where data from earlier scans were required for analyses, births were excluded if they occurred at the one site that only provided data from the last scan (missing data on all other scans), leaving a total sample of ≈15,305 across imputed datasets. Patterns were also stratified by the presence or absence of an indication for serial fetal ultrasound scans. This required restriction to the sample with complete data on comorbidities and antenatal complications. Pregnancies were additionally excluded if care occurred in sites that did not provide any data on PAPP-A, leaving a total sample size of ≈7,025 (Table 4).

Almost half of the pregnancies with unidentified SGA (47.1%) had no record of a fetal growth scan conducted at the site at which the women gave birth and 36.7% of women with unidentified SGA and an indication for serial screening did not undergo any scans. Over half (56.1%) of women who had SGA diagnosed antenatally required only one screening scan, meaning that EFW was below the 10^th^ centile at the time of the first scan. Few women with identified SGA required more than three scans before SGA was identified. Regardless of the presence of indication for serial scans, a lower proportion of women with unidentified SGA underwent screening every ≤3 or 4 weeks compared to women with identified SGA; 42.7% of women with identified SGA underwent screening scans with high frequency (every 3 weeks or more often). Screening scans were generally commenced slightly later for women with unidentified SGA compared to those with identified SGA, with a lower proportion commencing scans before 31 weeks’ in the former group (46.3% *vs* 56.3%). The patterns for women with or without a documented indication for serial scans were similar to the unstratified results, although a higher proportion of women with a scan indication underwent scans, and conversely, more women without a documented scan indication did not undergo any scans. More women with a scan indication commenced their scans before 31 weeks (59.0% if SGA was unidentified, 70.0% if SGA was identified) (Table 4).

**Figure 2:**
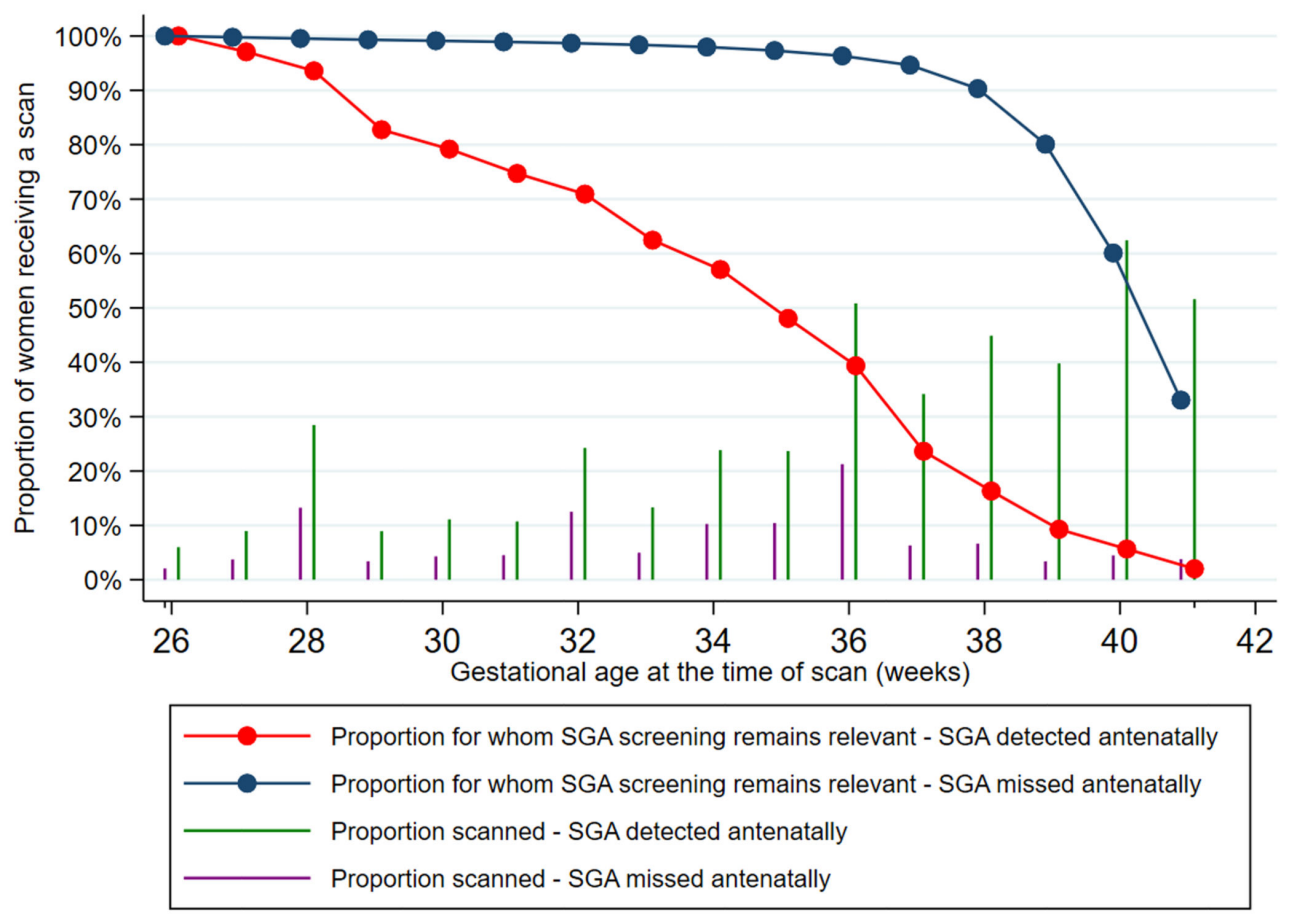
Proportion of women receiving a screening ultrasound for fetal growth, amongst the proportion in whom screening for SGA remains relevant, presented by SGA detection status.

For pregnancies in which screening for SGA remained relevant (pregnancy ongoing and SGA had not yet been identified), the proportion of women undergoing any ultrasound scan during each gestational week starting from 26 weeks is presented in Figure 2. Screening ultrasound scans remained applicable to over 90% of women with unidentified SGA until 37 weeks’, after which the proportion of women for whom it remained applicable decreased as the babies were born. In cases in which SGA was identified antenatally, the gestational age of the initial diagnosis was evenly distributed throughout the third trimester. This was demonstrated by a linear decrease in the proportion of women receiving screening scans across the gestational ages. Amongst pregnancies in which SGA was not identified, screening scans were less common at all gestational ages when compared to women with identified SGA. Despite screening scans remaining relevant to a larger proportion of pregnancies at term amongst women with unidentified SGA vs those with identified SGA, fewer than 10% of remaining women underwent a scan during any week of gestation at term.

Women with unidentified SGA had an adjusted mean of 18.0 additional days between their last scan and delivery compared to women with identified SGA (28.2 days *vs* 10.5 days; adjusted difference, 18.0 (95% CI, 17.2–18.8) days, *P <* 0.001); this is partly because many of the women with identified SGA underwent surveillance scans (no longer requiring screening) for diagnosed SGA. The mean duration between the last scan and birth increased with increasing gestational age at birth; pregnancies in which SGA was not identified had the last scan conducted 30.7 days (SD 21.7) before birth if birth occurred at or after 39+0 weeks and 18.7 (SD 16.4) days before birth if it occurred between 37+0 and 38+6 weeks.

Of all SGA babies, 90.3% were born at term. The results of the analysis limited to these cases focusing on EFW and EFW centiles at the last ultrasound scan before birth compared with birth weight and birth-weight centiles are reported in Table 5. The 13.3% of unidentified SGA babies born within a week of the last growth scan had a mean EFW centile of 25.6 (SD: 14.0). The difference between EFW and birth-weight centiles in the unidentified SGA group was 19.5 (SD: 13.8) centiles, with an adjusted mean difference in difference between centiles compared with the identified SGA group of 19.0 (95% CI, 17.8–20.1) centiles (*P <* 0.01). The difference between EFW and birth weight in g expressed as a percentage of birth weight in the unidentified SGA group was 13.5% (SD: 7.3%), with an adjusted mean difference in percentage difference compared with the identified SGA group of 9.8% (95% CI, 9.0–10.6%) (*P <* 0.01). As the duration between the last growth ultrasound scan and birth increased, the centile difference in the identified SGA group increased only marginally, although the difference between EFW at the time of scan and the actual birth weight a few weeks later increased, as expected. For pregnancies in which SGA was not identified antenatally, a different relationship was seen. For these pregnancies, as the duration between the last scan and birth increased, the difference between centiles increased, but the percentage difference between EFW and birth weight decreased; thus, EFW measurements taken 4 weeks before birth were closer to the actual birth weight than EFW measurements taken within 1 week of birth (difference of -3.0% (SD: 9.2%) for scans 3-4 weeks before birth and 13.5% (SD: 7.3%) for scans within 1 week of birth).

### Sensitivity analysis

On available case sensitivity analysis, characteristics and comparisons for the included SGA pregnancies were broadly similar to the main analysis, with consistent point estimates for all studied characteristics and patterns of ultrasound use, except for pre-existing diabetes (aOR 0.9 (95% CI, 0.5–1.5), p=0.64), which was no longer associated with SGA detection status. Whilst there was a loss of statistical significance at *P <* 0.05 threshold, this is very likely to be due to loss in statistical power from the reduced sample size (Tables A-D, Appendix S1).

The sensitivity analysis was also conducted after restricting the sample to pregnancies with a recorded anomaly scan at the site of birth. Fewer women underwent a presumed anomaly scan at the cluster site in which they later gave birth in the unidentified SGA group compared to the identified SGA group (76.1% vs 90.8%). The rate of detection of SGA (24.4%) in this restricted sample was similar to that in the main analysis. Compared with the primary sample, the sample restricted to pregnancies with an anomaly scan showed similar findings (Tables A-B, Appendix S2), except that an additional association was found, whereby the risk of unidentified SGA was lower amongst women with pre-existing hypertension (aOR 0.6 (95% CI, 0.4–0.9), *P <* 0.01). With regard to the patterns of ultrasound use, a lower proportion of women with unidentified SGA underwent no fetal growth scan after 24+0 weeks of pregnancy (36.6% of all women). All other findings were similar (Tables C-D, Appendix S2).

## Discussion

### Summary of key findings

Overall, 78.7% of SGA cases were missed antenatally. Having no recorded indication for serial ultrasound increased the risk of missing SGA antenatally; 68.5% of all SGA pregnancies had no known indication. Almost half of pregnancies with unidentified SGA had no growth scan, despite one-third of them having an indication. Non-cephalic presentation also reduced the chance of unidentified SGA, but BMI of 25.0–29.9 kg/m^2^ and less severe SGA increased the risk. For women with unidentified SGA who underwent growth scans, the last scan-to-birth interval widened with later birth, demonstrating policies to stop scanning at 36 weeks. EFW from scans conducted within a week before birth was overestimated by 10.3 centiles for all SGA term babies and by more amongst unidentified SGA babies (19.5 centiles).

### Interpretation of findings

Whilst we expected that having an indication for serial scans would increase SGA detection (demonstrating the application of national targeted screening),^29^ it is less established that most SGA pregnancies have no risk factors. Such women are deprioritized and undergo less sensitive screening (fundal height measurement),^30, 31^ increasing their risk of unidentified SGA. Amongst women at low risk of SGA, serial scans also have low sensitivity, presenting a diagnostic challenge.^12, 13^ Furthermore, it is not clear whether the unidentified SGA babies born to women with no SGA risk factors have the same risk of adverse outcome as SGA babies born to women with predisposing factors.

Over half of cases with unidentified SGA were born after 40 weeks, some of which had earlier growth scans demonstrating normal size. Unidentified SGA in this context can be explained by late-onset growth restriction, overestimated fetal weight, loss of fetal weight or a combination of these. Overestimated fetal weight has been reported previously by a meta-analysis,^32^ and fetal weight loss has been hypothesized in studies focusing on London and multicenter cohorts, which reported similar rates of SGA detection to those reported here.^33 34^

The reduced risk of unidentified SGA in non-cephalic cases may be explained by incidental SGA identification when scanning for suspected non-cephalic presentation.^35^ A UK report recommending universal late pregnancy ultrasound screening for fetal presentation, but without simultaneous fetal growth assessment (as is often practiced), did not consider whether this will reduce SGA detection amongst non-cephalic babies^36^.

Women with BMI of 25.0–29.9 kg/m2 (and possibly with BMI of 30.0–34.9 kg/m2) were at greater risk of unidentified SGA compared to those with healthy BMI. Fundal height measurement is affected by maternal BMI, although current protocols recommend serial ultrasound only for women with BMI above 35 kg/m^2^.^31, 37–39^ Given the considerable proportion of women with unidentified SGA who had a BMI of 25.0–29.9 kg/m^2^ (27.1%), research investigating methods to improve fetal weight estimation in this group is expected to have wide impact.

### Strengths and limitations

To the best of our knowledge, this is the largest and most comprehensive study on this topic, suggesting novel targets to improve SGA screening.^19, 20, 40^ The use of data from electronic patient records allowed inclusion of a large sample but was limited by data quality and availability.^24^ The analysis assessed detection amongst SGA neonates, although we are aware that FGR is better correlated with risk of perinatal morbidity and mortality and therefore may be a better screening target to reduce the rate of adverse outcome and limit iatrogenic harm. Nevertheless, detection of SGA is the end target of national and international guidelines on this topic,^10^ hence our decision to define our primary outcome in this way. Data were not available on some indications for serial fetal ultrasound in the UK,^41, 42^ although the missing indicators were either rare (e.g. chronic kidney disease) or could have affected only the 42.2% of women with no known risk factor who were multiparous (no data on previous stillbirth or SGA pregnancy). Our assumption that women with no ultrasound record had no scans had little impact when tested on sensitivity analysis. The results are generalizable to maternity care settings in the UK and other countries that adopt similar selective ultrasound strategies for fetal growth.^31, 41^

### Implication of findings

Given the proportion of women who underwent no serial scans despite having an indication, investigating missed cases of SGA is key to improving care quality. Maternity units in the DESiGN trial cited resource availability (including sonographer shortages) as a reason for incomplete concordance with national guidelines on SGA screening.^43^ Economic evaluations assessing the strategy of offering serial ultrasound to women with less implemented indications, such as BMI 35–40 kg/m^2^, are required to demonstrate the cost-effectiveness of recommended practice.

Further research is also required to assess alternative screening strategies for women without known risk factors for SGA. Whilst performing a single growth ultrasound scan has only low to moderate sensitivity in this group, the sensitivity improves with advancing gestation.^30, 32^ The optimal timing of a universally offered late scan and the effect of measuring the change in the EFW centile between two scans for women who have a one-off indication for a fetal scan (e.g. small fundal height measurement) are unknown. Policies to continue serial scans until birth have been introduced into common UK practice through the Saving Babies Lives care bundle^29, 44^ but were not widely implemented in the studied maternity units. There is currently no published research studying the benefit of this resource-intense policy, except when part of complex interventions.^45–47^ Studies of the accuracy of ultrasound assessment of EFW at term vary in their findings,^48–51^ but accuracy appears to be problematic; techniques are required to improve this strategy and other methods (e.g. biomarkers of placental function) that identify the fetus at risk of perinatal mortality.

## Conclusions

The risk of antenatally unidentified SGA is greater in the absence of indication for serial ultrasound growth scans, with BMI between 25.0–29.9 kg/m^2^, less severe SGA and cephalic presentation. Two-thirds of pregnancies with SGA had no indication for serial growth scans, emphasizing the need to improve SGA screening in low-risk populations. Amongst those who underwent a scan, the EFW was generally overestimated, precluding SGA diagnosis.

Missed case analysis should play an important role in quality improvement. Further research is needed to determine how SGA detection can be improved for women who are overweight or without classic risk factors for SGA and identify which of the unidentified SGA cases are most at risk of adverse outcome.

## Supplementary Material

Appendix 1

Appendix 2

Appendix 3

## Figures and Tables

**Table 1:** Maternal and perinatal characteristics of included pregnancies, according to whether small-for-gestational-age (SGA) fetus was identified antenatally*

Characteristic		Unidentified SGA (n ≈ 7532)	Identified SGA (n ≈ 1878)
**Age (years)**		30.5 (5.5)	30.9 (5.7)
	**≤ 40 years**	96.3	94.8
	**>40 years**	3.7	5.2
**IMD quintile**			
	**1** (**least deprived**)	9.0	10.8
	**2**	11.7	12.7
	**3**	24.8	23.6
	**4**	35.6	33.4
	**5** (**most deprived**)	19.0	19.6
**Race**			
	**White**	38.2	36.5
	**Black**	16.9	17.0
	**Asian**	31.8	33.8
	**Mixed**	1.7	2.0
	**Other**	11.4	10.6
**BMI (kg/m^2^) **		25.0 (5.2)	24.8 (5.6)
	**<18.5**	5.0	7.5
	**18.5–24.9 **	52.9	52.2
	**25.0–29.9 **	27.1	25.0
	**30.0–34.9**	10.0	9.9
	**35.0–39.9**	3.4	3.6
	**≥** **40.0**	1.6	1.9
**Parity**			
	**0**	59.4	56.9
	**1**	25.5	27.7
	**2**	9.2	9.1
	**3**	3.4	3.9
	**≥** **4 **	2.5	2.4
**Smoker**		8.7	10.4
**Comorbidity**			
	**Hypertension**	1.9	3.0
	**Diabetes**	1.2	2.1
**Antenatal complication**			
	**Pre-eclampsia**	2.6	6.9
	**Gestational hypertension**	1.8	3.6
	**Gestational diabetes**	4.6	6.8
**PAPP-A**			
	**<0.300MoM**	1.6	4.6
	**0.3–0.415MoM**	3.0	6.6
**>0.415MoM**		45.5	50.1
	**Missing data**	49.8	38.7
**Any indication for serial fetal scans **		28.5	42.8
**Cephalic presentation at birth**		95.3	91.2
**Gestational age at birth (weeks)**		37.7 (3.0)	39.8 (2.4)
	**<28+0 **	0.8	1.9
	**28+0–33+6 **	1.8	8.0
	**34+0–36+6 **	3.3	15.2
	**37+0–37+6 **	4.4	15.3
	**38+0–38+6 **	9.4	19.8
	**39+0–39+6 **	19.3	17.6
	**≥** **40+0 **	61.0	22.2
**Birth-weight centile**		5.4 (2.9)	4.0 (2.8)
	**<3^rd^ centile **	24.9	43.5
	**3^rd^–5^th^ centile **	18.7	20.9
	**5^th^-10^th^ centile **	56.5	35.5

* Data are given as % or mean (SD). Complete case data except that information on PAPP-A may be missing. Data using multiply imputed datasets provides only percentages of characteristics of interest. Previous reports have reported demographic characteristics of the trial population.^21^

**Table 2:** Association of maternal characteristics with unidentified small-for-gestational age (SGA)*

Characteristic		Unidentified SGA ( n ≈7532)	Identified SGA ( n ≈ 1878)	Unadjusted OR (95% CI)	Adjusted† OR (95% CI)	Adjusted *P*†
**Age**						
	**≤** **40 years**	80.4	19.6	Ref	Ref	0.03
	**>40 years**	74.1	25.9	0.69 (0.53–0.90)	0.74 (0.56–0.98)	
**IMD quintile**						0.47
	**1 (least deprived)**	77.0	23.0	Ref	Ref	0.47
	**2**	78.8	21.2	1.10 (0.88–1.36)	0.97 (0.77–1.23)	
	**3**	81.0	19.0	1.28 (1.05–1.54)	1.14 (0.92–1.41)	
	**4**	81.2	18.8	1.27 (1.06–1.53)	1.10 (0.89–1.35)	
	**5 (most deprived)**	79.7	20.3	1.14 (0.94–1.39)	1.05 (0.83–1.32)	
**Race**						
	**White**	80.9	19.1	Ref	Ref	<0.01
	**Black**	80.1	19.9	0.95 (0.82–1.11)	0.95 (0.80–1.13)	
	**Asian**	79.2	20.8	0.92 (0.81–1.04)	0.80 (0.69–0.93)	
	**Mixed**	77.2	22.8	0.80 (0.54–1.19)	0.86 (0.57–1.31)	
	**Other**	81.3	18.7	1.03 (0.85–1.24)	0.81 (0.65–1.00)	
**BMI (kg/m^2^)**						
	**<18.5 kg/m^2^**	73.1	26.9	0.63 (0.51–0.79)	0.61 (0.48–0.76)	0.04
	**18.5–24.9 kg/m^2^**	80.4	19.6	Ref	Ref	
	**25.0–29.9 kg/m^2^**	81.5	18.5	1.07 (0.94–1.22)	1.15 (1.01–1.32)	
	**30.0–34.9 kg/m^2^**	80.3	19.7	1.00 (0.83–1.21)	1.12 (0.91–1.38)	
	**35.0–39.9 kg/m^2^**	79.2	20.8	0.93 (0.69–1.24)	1.04 (0.77–1.42)	
	**≥** **40.0 kg/m^2^**	77.5	22.5	0.82 (0.54–1.26)	0.99 (0.63–1.54)	
**Parity**						
	**0**	80.9	19.1	Ref	Ref	0.15
	**1**	78.8	21.2	0.90 (0.80–1.02)	0.85 (0.74–0.97)	
	**2**	80.4	19.6	1.00 (0.83–1.22)	0.99 (0.80–1.22)	
	**3**	77.6	22.4	0.82 (0.62–1.09)	0.83 (0.62–1.12)	
	**≥4**	80.9	19.1	0.99 (0.69–1.42)	1.00 (0.67–1.48)	
**Smoking status**						
	**Non-smoker**	80.5	19.5	Ref	Ref	Ref
	**Smoker**	77.2	22.8	0.82 (0.69–0.97)	0.79 (0.66–0.96)	0.02

* Data are given as %, unless stated otherwise. Data using multiply imputed datasets provides only percentages of characteristics of interest.

† Adjusted for all other demographic and clinical characteristics (age, index of socioeconomic deprivation (IMD) quintile, race, body mass index (BMI), parity and smoking status), birth-weight centile, maternal comorbidities and antenatal complications, and cluster site and trial phase.

**Table 3:** Association of comorbidities and fetal characteristics with unidentified small-for-gestational age (SGA)

Characteristic		Unidentified SGA ( n ≈7532)	Identified SGA ( n ≈ 1878)	Unadjusted OR (95% CI)	Adjusted OR (95% CI) †	Adjusted *P*†
**Comorbidity**						
	**No hypertension**	80.4	19.6	Ref	Ref	Ref
	**Hypertension**	71.6	28.4	0.62 (0.45–0.86)	0.83 (0.59–1.17)	0.29
	**No diabetes**	80.3	19.7	Ref	Ref	Ref
	**Diabetes**	69.3	30.7	0.51 (0.35–0.76)	0.52 (0.34–0.79)	<0.01
**Antenatal complication**						
	**No pre-eclampsia**	80.9	19.1	Ref	Ref	Ref
	**Pre-eclampsia**	60.4	39.6	0.34 (0.27–0.44)	0.40 (0.31–0.51)	<0.01
	**No gestational hypertension**	80.5	19.5	Ref	Ref	Ref
	**Gestational hypertension**	66.7	33.3	0.47 (0.34–0.63)	0.54 (0.39–0.74)	<0.01
	**No GDM**	80.6	19.4	Ref	Ref	Ref
	**GDM**	73.0	27.0	0.65 (0.52–0.80)	0.64 (0.51–0.80)	<0.01
**PAPP-A **						
	**<0.300 MoM**	57.3	42.7	0.38 (0.28–0.53)	0.45 (0.32–0.64)	<0.01
	**0.300–0.415MoM**	64.0	36.0	0.51 (0.39–0.66)	0.56 (0.43–0.75)	
	**>0.415MoM**	77.8	22.2	Ref	Ref	Ref
Indication for serial fetal scans*						
	**No indication**	82.8	17.2	Ref	Ref	Ref
	**Any indication**	72.0	28.0	0.53 (0.47–0.60)	0.56 (0.49–0.64)‡	<0.01
**Neonatal presentation at birth **						
** Cephalic**		81.4	18.6	Ref	Ref	Ref
	**Non-cephalic**	69.2	30.8	0.49 (0.39–0.61)	0.58 (0.46–0.73)	<0.01
**B** **irth-weight centile§**		5.4 (2.9)	4.0 (2.8)	1.20§ (1.17–1.22)	1.21§ (1.18–1.23)	<0.01

* Data are given as % or mean (SD), unless stated otherwise. Available case data (except for data on PAPP-A, which was included even if missing).

† Odds ratio (OR) for unidentified SGA, adjusted for all other demographic and clinical characteristics (age, index of socioeconomic deprivation (IMD) quintile, race, body mass index (BMI), parity and smoking status), allocated birth-weight centile, maternal comorbidities and antenatal complications, and cluster site and trial phase.

‡ Adjusted only for IMD, parity, race and birth-weight centile (not for other characteristics, which are included in this composite).

§ Change in OR with one centile increase (<10^th^ centile).

**Table 4:** Patterns of ultrasound use for all small-for-gestational-age (SGA) pregnancies, stratified by presence or absence of a recorded indication for serial fetal growth scans

		All SGA (n ≈15,784)		SGA with serial scan indication		SGA with no recorded serial scan indication†	
Characteristic		Unidentified SGA (n ≈12,416)	Identified SGA (n ≈3,368)	Unidentified SGA (n ≈1,591)	Identified SGA (n ≈619)	Unidentified SGA (n ≈3,989)	Identified SGA (n ≈826)
**Number of screening scans performed**							
	**0**	47.1	-	36.7	-	55.1	-
	**1**	21.4	56.1	17.9	54.1	20.6	59.4
	**2**	14.8	26.5	19.9	25.6	12.2	25.4
	**3**	10.8	12.4	17.2	14.3	8.1	10.3
	**4**	4.1	4.2	5.6	4.7	2.9	4.3
	**≥** **5**	1.8	0.8	2.6	1.4	1.0	0.6
Screening scan frequency*							
	**≤** **3-weekly**	14.5	42.7	14.2	43.1	15.1	42.8
	**4-weekly**	14.0	30.0	12.2	26.2	13.3	25.9
	**>4-weekly**	71.6	27.3	73.6	30.7	71.6	31.3
**GA at first scan (if scans conducted)**							
	**<31+0**	46.3	56.3	59.0	70.0	42.3	47.3
	**31+0–33+6**	15.6	13.7	14.8	11.9	15.6	16.1
	**34+0–36+6**	27.4	19.7	20.3	13.0	26.2	22.5
	**≥** **37+0**	10.7	10.3	5.9	5.1	15.9	14.2

* Data are given as %. Data using multiply imputed datasets provides only percentages of characteristics of interest. Analysis according to presence of serial scan indication was restricted to the sample with complete data on comorbidities and antenatal complications from sites that provided data on PAPP-A. For pregnancies with at least two scans.

† Includes records for which PAPP-A was not documented.

**Table 5:** Comparison of estimated fetal weight at the last ultrasound scan and the birth weight, including their centiles, for small-for-gestational-age (SGA) babies born at term

			Unidentified SGA (n ≈5,544) _ _	Identified SGA (n ≈1,525)	Unadjusted mean difference (95% CI)	Adjusted mean difference (95% CI)*	Adjusted P*
**Last scan within 1 week of birth**							
	**EFW centile at last scan**		25.6 (14.0)	4.6 (2.9)	20.9 (19.8–22.0)	20.6 (19.5–21.7)	<0.01
	**Difference between EFW and birth-weight centiles**		19.5 (13.8)	0.2 (3.3)	19.3 (18.2–20.4)	19.0 (17.8–20.1)	<0.01
	**Percentage difference between EFW and birth weight**		13.5 (7.3)	2.4 (10.9)	11.0 (10.1–12.0)	9.8 (9.0–10.6)	<0.01
**Last scan within 1–2 weeks of birth**							
	**EFW centile at last scan**		26.8 (14.1)	5.3 (2.8)	21.5 (19.8–23.1)	21.2 (19.5–22.8)	<0.01
	**Difference between EFW and birth-weight centiles**		21.0 (14.0)	0.6 (3.4)	20.3 (18.7–21.9)	20.0 (18.4–21.7)	<0.01
	**Percentage difference between EFW and birth weight**		10.9 (38.0)	-2.6 (9.1)	13.5 (9.1–17.9)	12.9 (8.5–17.3)	<0.01
**If scan within 2–3 weeks ** **of birth**							
	**EFW centile at last scan**		27.1 (14.1)	5.4 (2.8)	21.7 (19.9–23.5)	21.5 (19.6–23.3)	<0.01
	**Difference between EFW and birth-weight centiles**		21.0 (14.0)	1.2 (3.6)	19.8 (18.0–21.6)	19.7 (17.8–21.5)	<0.01
	**Percentage difference between EFW and birth weight**		3.2 (27.1)	-8.1 (12.2)	11.2 (7.7–14.8)	9.2 (5.6–12.8)	<0.01
**Last scan within 3–4 weeks ** **of birth**							
	**EFW centile at last scan**		29.7 (15.0)	5.6 (3.3)	24.1 (21.6–26.6)	24.0 (21.4–26.6)	<0.01
	**Difference between EFW and birth-weight centiles**		24.0 (14.8)	1.6 (4.2)	22.3 (19.9–24.7)	22.1 (19.5–24.6)	<0.01
	**Percentage difference between EFW and birth weight**		-3.0 (9.2)	-13.2 (27.2)	10.2 (7.9–12.6)	5.6 (3.4–7.9)	<0.01

* Data are given as mean (SD), unless stated otherwise. Differences are calculated by minusing the birthweight (weight/centile) from the EFW (weight/centile). Adjusted for cluster site and trial phase only.

## References

[1] Every Woman Every Child: The Global Strategy for Women’s (2016-2030). Children’s and Adolescents’ Health.

[2] Gardosi J, Kady SM, McGeown P, Francis A, Tonks A (2005). Classification of stillbirth by relevant condition at death (ReCoDe): population based cohort study. BMJ.

[3] Flenady V, Middleton P, Smith GC, Duke W, Erwich JJ, Khong TY, Neilson J, Ezzati M, Koopmans L, Ellwood D, Fretts R (2011). Stillbirths: the way forward in high-income countries. Lancet.

[4] Gardosi J, Madurasinghe V, Williams M, Malik A, Francis A (2013). Maternal and fetal risk factors for stillbirth: population based study. BMJ.

[5] Froen JF, Gardosi JO, Thurmann A, Francis A, Stray-Pedersen B (2004). Restricted fetal growth in sudden intrauterine unexplained death. Acta Obstet Gynecol Scand.

[6] Efkarpidis S, Alexopoulos E, Kean L, Liu D, Fay T (2004). Case-control study of factors associated with intrauterine fetal deaths. MedGenMed.

[7] Poon LC, Volpe N, Muto B, Syngelaki A, Nicolaides KH (2012). Birthweight with gestation and maternal characteristics in live births and stillbirths. Fetal Diagn Ther.

[8] Iliodromiti S, Mackay DF, Smith GC, Pell JP, Sattar N, Lawlor DA, Nelson SM (2017). Customised and Noncustomised Birth Weight Centiles and Prediction of Stillbirth and Infant Mortality and Morbidity: A Cohort Study of 979,912 Term Singleton Pregnancies in Scotland. PLoS Med.

[9] Flenady V, Wojcieszek AM, Middleton P, Ellwood D, Erwich JJ, Coory M, Khong TY, Silver RM, Smith GC, Boyle FM, Lawn JE (2016). Stillbirths: recall to action in high-income countries. Lancet.

[10] McCowan LM, Figueras F, Anderson NH (2018). Evidence-based national guidelines for the management of suspected fetal growth restriction: comparison, consensus, and controversy. Am J Obstet Gynecol.

[11] Backe B, Nakling J (1993). Effectiveness of antenatal care: a population based study. Br J Obstet Gynaecol.

[12] Monier I, Blondel B, Ego A, Kaminiski M, Goffinet F, Zeitlin J (2015). Poor effectiveness of antenatal detection of fetal growth restriction and consequences for obstetric management and neonatal outcomes: a French national study. BJOG.

[13] Jahn A, Razum O, Berle P (1998). Routine screening for intrauterine growth retardation in Germany: low sensitivity and questionable benefit for diagnosed cases. Acta Obstet Gynecol Scand.

[14] Mattioli KP, Sanderson M, Chauhan SP (2010). Inadequate identification of small-for-gestational-age fetuses at an urban teaching hospital. Int J Gynaecol Obstet.

[15] Kean L, Liu D (1996). Antenatal care as a screening tool for the detection of small for gestational age babies in the low risk population. Journal of Obstetrics & Gynaecology.

[16] Chauhan SP, Beydoun H, Chang E, Sandlin AT, Dahlke JD, Igwe E, Magann EF, Anderson KR, Abuhamad AZ, Ananth CV (2014). Prenatal detection of fetal growth restriction in newborns classified as small for gestational age: correlates and risk of neonatal morbidity. Am J Perinatol.

[17] Fratelli N, Valcamonico A, Prefumo F, Pagani G, Guarneri T, Frusca T (2013). Effects of antenatal recognition and follow-up on perinatal outcomes in small-for-gestational age infants delivered after 36 weeks. Acta Obstet Gynecol Scand.

[18] Lindqvist PG, Molin J (2005). Does antenatal identification of small-for-gestational age fetuses significantly improve their outcome?. Ultrasound Obstet Gynecol.

[19] Andreasen LA, Tabor A, Norgaard LN, Taksoe-Vester CA, Krebs L, Jorgensen FS, Jepsen IE, Sharif H, Zingenberg H, Rosthoj S, Sorensen AL (2021). Why we succeed and fail in detecting fetal growth restriction: A population-based study. Acta Obstet Gynecol Scand.

[20] Diksha P, Permezel M, Pritchard N (2018). Why we miss fetal growth restriction: Identification of risk factors for severely growth-restricted fetuses remaining undelivered by 40 weeks gestation. Aust N Z J Obstet Gynaecol.

[21] Vieira MC, Relph S, Muruet-Gutierrez W, Elstad M, Coker B, Moitt N, Delaney L, Winsloe C, Healey A, Coxon K, Alagna A (2022). Evaluation of the Growth Assessment Protocol (GAP) for antenatal detection of small for gestational age: The DESiGN cluster randomised trial. PLoS Med.

[22] Relph S, Vieira MC, Copas A, Coxon K, Alagna A, Briley A, Johnson M, Page L, Peebles D, Shennan A, Thilaganathan B (2022). Improving antenatal detection of small-for-gestational-age fetus: economic evaluation of Growth Assessment Protocol. Ultrasound Obstet Gynecol.

[23] Vieira MC, Relph S, Copas A, Healey A, Coxon K, Alagna A, Briley A, Johnson M, Lawlor DA, Lees C, Marlow N (2019). The DESiGN trial (DEtection of Small for Gestational age Neonate), evaluating the effect of the Growth Assessment Protocol (GAP): study protocol for a randomised controlled trial. Trials.

[24] Relph S, Elstad M, Coker B, Vieira MC, Moitt N, Gutierrez WM, Khalil A, Sandall J, Copas A, Lawlor DA, Pasupathy D (2021). Using electronic patient records to assess the effect of a complex antenatal intervention in a cluster randomised controlled trial-data management experience from the DESiGN Trial team. Trials.

[25] Cole TJ, Freeman JV, Preece MA (1998). British 1990 growth reference centiles for weight, height, body mass index and head circumference fitted by maximum penalized likelihood. Stat Med.

[26] Pinnock H, Barwick M, Carpenter CR, Eldridge S, Grandes G, Griffiths CJ, Rycroft-Malone J, Meissner P, Murray E, Patel A, Sheikh A (2017). Standards for Reporting Implementation Studies (StaRI) Statement. BMJ.

[27] Hadlock FP, Harrist RB, Martinez-Poyer J (1991). In utero analysis of fetal growth: a sonographic weight standard. Radiology.

[28] Rubin D (1987). Multiple imputation for nonresponse in surveys.

[29] NHS England Saving Babies’ Lives Version Two.

[30] Goto E (2013). Prediction of low birthweight and small for gestational age from symphysis-fundal height mainly in developing countries: a meta-analysis. Journal of Epidemiology and Community Health.

[31] Royal College of Obstetricians & Gynaecologists Small for Gestational Age Fetus: Investigation & Management Green-top Guideline No 31.

[32] Caradeux J, Martinez-Portilla RJ, Peguero A, Sotiriadis A, Figueras F (2019). Diagnostic performance of third-trimester ultrasound for the prediction of late-onset fetal growth restriction: a systematic review and meta-analysis. Am J Obstet Gynecol.

[33] Stampalija T, Wolf H, Mylrea-Foley B, Marlow N, Stephens KJ, Shaw CJ, Lees CC (2022). Reduced fetal growth velocity and weight loss are associated with adverse perinatal outcome in fetuses at risk of growth restriction. Am J Obstet Gynecol.

[34] Stephens K, Al-Memar M, Beattie-Jones S, Dhanjal M, Mappouridou S, Thorne E, Lees C (2019). Comparing the relation between ultrasound-estimated fetal weight and birthweight in cohort of small-for-gestational-age fetuses. Acta Obstet Gynecol Scand.

[35] National Institute for Health and Care Excellence Antenatal care for uncomplicated pregnancies.

[36] Smith GC, Moraitis AA, Wastlund D, Thornton JG, Papageorghiou A, Sanders J, Heazell AE, Robson SC, Sovio U, Brocklehurst P, Wilson EC (2021). Universal late pregnancy ultrasound screening to predict adverse outcomes in nulliparous women: a systematic review and cost-effectiveness analysis. Health Technol Assess.

[37] Preyer O, Husslein H, Concin N, Ridder A, Musielak M, Pfeifer C, Oberaigner W, Husslein P (2019). Fetal weight estimation at term - ultrasound versus clinical examination with Leopold’s manoeuvres: a prospective blinded observational study. BMC Pregnancy Childbirth.

[38] Fox NS, Rebarber A, Silverstein M, Roman AS, Klauser CK, Saltzman DH (2013). The effectiveness of antepartum surveillance in reducing the risk of stillbirth in patients with advanced maternal age. Eur J Obstet Gynecol Reprod Biol.

[39] Aksoy H, Aksoy U, Karadag OI, Yucel B, Aydin T, Babayigit MA (2015). Influence of maternal body mass index on sonographic fetal weight estimation prior to scheduled delivery. J Obstet Gynaecol Res.

[40] Kajdy A, Modzelewski J, Jakubiak M, Pokropek A, Rabijewski M (2019). Effect of antenatal detection of small-for-gestational-age newborns in a risk stratified retrospective cohort. PLoS One.

[41] O’Conner D (2016). Saving Babies’ Lives: A care bundle for reducing stillbirth.

[42] National Institute for Health and Care Excellence Hypertension in pregnancy: diagnosis and management.

[43] Relph S, Coxon K, Vieira M, Copas A, Healey A, Alagna A, Briley A, Johnson M, Lawlor D, Lees C, Marlow N (2022). Effect of the Growth Assessment Protocol on the DEtection of the Small for GestatioNal Age Fetus: Process evaluation from the DESiGN cluster randomised trial. Implementation Science.

[44] Williams M, Turner S, Butler E, Gardosi J (2018). Fetal growth surveillance - Current guidelines, practices and challenges. Ultrasound.

[45] Hugh O, Williams M, Turner S, Gardosi J (2021). Reduction of stillbirths in England from 2008 to 2017 according to uptake of the Growth Assessment Protocol: 10-year population-based cohort study. Ultrasound Obstet Gynecol.

[46] Jayawardena L, Sheehan P (2019). Introduction of a customised growth chart protocol increased detection of small for gestational age pregnancies in a tertiary Melbourne hospital. Aust N Z J Obstet Gynaecol.

[47] Cowan FJ, McKinlay CJD, Taylor RS, Wilson J, McAra-Couper J, Garrett N, O’Brien A, McCowan LME (2021). Detection of small for gestational age babies and perinatal outcomes following implementation of the Growth Assessment Protocol at a New Zealand tertiary facility: An observational intervention study. Aust N Z J Obstet Gynaecol.

[48] Stubert J, Peschel A, Bolz M, Glass A, Gerber B (2018). Accuracy of immediate antepartum ultrasound estimated fetal weight and its impact on mode of delivery and outcome - a cohort analysis. BMC Pregnancy Childbirth.

[49] Francis A, Tonks A, Gardosi J (2011). Accuracy of ultrasound estimation of fetal weight at term. Archives of Disease in Childhood - Fetal and Neonatal Edition.

[50] Milner J, Arezina J (2018). The accuracy of ultrasound estimation of fetal weight in comparison to birth weight: A systematic review. Ultrasound.

[51] Castro-Vasquez BA, Taboada C (2020). Accuracy of Estimated Fetal Weight in Third Trimester [33A]. Obstetrics & Gynecology.

