# Supplementary material for: Characteristics associated with antenatally unidentified small-for-gestational-age fetuses: prospective cohort study nested within the DESiGN randomized control trial
Source: Ultrasound Obstet Gynecol. Author manuscript; Available in PMC 2024 Jun 3. (PMC7616055; doi:10.1002/uog.26091)
Supplement: Appendix 1 [file EMS196131-supplement-Appendix_1.docx]

STROBE Statement—checklist of items that should be included in reports of observational studies

|  | Item No. | Recommendation | Page  No. | Relevant text from manuscript |
| --- | --- | --- | --- | --- |
| **Title and abstract** | 1 | (*a*) Indicate the study’s design with a commonly used term in the title or the abstract | 1 | ‘a prospective cohort study’ |
|  |  | (*b*) Provide in the abstract an informative and balanced summary of what was done and what was found | 4 | Section ‘Abstract’ |
| Introduction | | | |  |
| Background/rationale | 2 | Explain the scientific background and rationale for the investigation being reported | 6 | Section ‘Introduction’ |
| Objectives | 3 | State specific objectives, including any prespecified hypotheses | 6 | This analysis aimed to identify the clinical characteristics and patterns of ultrasound use amongst pregnancies in which SGA is not identified antenatally, compared to those in which it is identified, to understand how we can better design interventions to improve detection. |
| Methods | | | |  |
| Study design | 4 | Present key elements of study design early in the paper | 7 | Section ‘Study Design’ |
| Setting | 5 | Describe the setting, locations, and relevant dates, including periods of recruitment, exposure, follow-up, and data collection | 7 | ‘a UK randomised cluster control trial conducted between 05/11/2016-28/3/2019’.  See also references 16-18 |
| Participants | 6 | (*a*) *Cohort study*—Give the eligibility criteria, and the sources and methods of selection of participants. Describe methods of follow-up  *Case-control study*—Give the eligibility criteria, and the sources and methods of case ascertainment and control selection. Give the rationale for the choice of cases and controls  *Cross-sectional study*—Give the eligibility criteria, and the sources and methods of selection of participants | 7 | ‘For this analysis, only pregnancies in which the baby was born SGA (defined as birthweight below the 10th centile for gestational age on population reference charts)19 after 24+0 gestational weeks and that were not exposed to the intervention were included (all pregnancies from control clusters and any pregnancies in intervention clusters that occurred prior to the implementation of GAP). Multiple pregnancies (i.e., twins) and those with antenatally-diagnosed fetal abnormalities were excluded. Women and babies in whom SGA detection status could not be determined because data on ultrasound were missing during an entire trial phase at a cluster site (affecting data from two clusters) were also excluded.’ |
|  |  | (*b*) *Cohort study*—For matched studies, give matching criteria and number of exposed and unexposed  *Case-control study*—For matched studies, give matching criteria and the number of controls per case | N/A |  |
| Variables | 7 | Clearly define all outcomes, exposures, predictors, potential confounders, and effect modifiers. Give diagnostic criteria, if applicable | 7-8 | Sections ‘Defining antenatally identified and unidentified cases of SGA’ and ‘Exposures’. |
| Data sources/ measurement | 8* | For each variable of interest, give sources of data and details of methods of assessment (measurement). Describe comparability of assessment methods if there is more than one group | References 16-18 |  |
| Bias | 9 | Describe any efforts to address potential sources of bias | 8-10 | Sections ‘Management of missing data’ and ‘Sensitivity Analyses’ |
| Study size | 10 | Explain how the study size was arrived at | Reference 16 |  |

Continued on next page

| Quantitative variables | 11 | Explain how quantitative variables were handled in the analyses. If applicable, describe which groupings were chosen and why | 8 | ‘Categories were chosen according to those used in routine clinical practice, including existing risk stratification models’ |
| --- | --- | --- | --- | --- |
| Statistical methods | 12 | (*a*) Describe all statistical methods, including those used to control for confounding | 9 | Section ‘Statistical analysis’ |
|  |  | (*b*) Describe any methods used to examine subgroups and interactions | 9 | Section ‘Statistical analysis’ |
|  |  | (*c*) Explain how missing data were addressed | 8-9 | Section ‘Management of missing data’ |
|  |  | (*d*) *Cohort study*—If applicable, explain how loss to follow-up was addressed  *Case-control study*—If applicable, explain how matching of cases and controls was addressed  *Cross-sectional study*—If applicable, describe analytical methods taking account of sampling strategy | N/A |  |
|  |  | (*e*) Describe any sensitivity analyses | 9-10 | Section ‘Sensitivity analyses’ |
| Results | | | | |
| Participants | 13* | (a) Report numbers of individuals at each stage of study—eg numbers potentially eligible, examined for eligibility, confirmed eligible, included in the study, completing follow-up, and analysed | 11 & Figure 1 | ‘Of the 169,724 pregnancies included in the control arm of the DESIGN RCT, 9.3% (n=15,784) were SGA at birth and included in the main analyses of this paper. […] Following exclusion of pregnancies with missing data on maternal co-morbidities and antenatal complications, 9,410 pregnancies were available for assessment of maternal and fetal characteristics associated with unidentified SGA (Figure 1).’ |
|  |  | (b) Give reasons for non-participation at each stage | N/A |  |
|  |  | (c) Consider use of a flow diagram | Figure 1 |  |
| Descriptive data | 14* | (a) Give characteristics of study participants (eg demographic, clinical, social) and information on exposures and potential confounders | Table 1 |  |
|  |  | (b) Indicate number of participants with missing data for each variable of interest | Appendix 2 |  |
|  |  | (c) *Cohort study*—Summarise follow-up time (eg, average and total amount) | N/A |  |
| Outcome data | 15* | *Cohort study*—Report numbers of outcome events or summary measures over time |  |  |
|  |  | *Case-control study—*Report numbers in each exposure category, or summary measures of exposure |  |  |
|  |  | *Cross-sectional study—*Report numbers of outcome events or summary measures | 11 | ‘Of these, SGA was not identified antenatally in 12,416 (78.7%).’ |
| Main results | 16 | (*a*) Give unadjusted estimates and, if applicable, confounder-adjusted estimates and their precision (eg, 95% confidence interval). Make clear which confounders were adjusted for and why they were included | Table 2, 3 and 5 |  |
|  |  | (*b*) Report category boundaries when continuous variables were categorized | Table 2, 3 and 5 |  |
|  |  | (*c*) If relevant, consider translating estimates of relative risk into absolute risk for a meaningful time period | N/A |  |

Continued on next page

| Other analyses | 17 | Report other analyses done—eg analyses of subgroups and interactions, and sensitivity analyses | 21, Appendices 2 & 3 |  |
| --- | --- | --- | --- | --- |
| Discussion | | | | |
| Key results | 18 | Summarise key results with reference to study objectives | 22 | Section ‘Summary of the key findings’ |
| Limitations | 19 | Discuss limitations of the study, taking into account sources of potential bias or imprecision. Discuss both direction and magnitude of any potential bias | 23 | Section ‘Strengths and limitations’ |
| Interpretation | 20 | Give a cautious overall interpretation of results considering objectives, limitations, multiplicity of analyses, results from similar studies, and other relevant evidence | 22-23 | Section ‘Interpretation of the findings’ |
| Generalisability | 21 | Discuss the generalisability (external validity) of the study results | 23 | ‘The results are generalisable to maternity care settings in the UK and other countries that adopt similar selective ultrasound strategies for fetal growth.’ |
| Other information | |  | | |
| Funding | 22 | Give the source of funding and the role of the funders for the present study and, if applicable, for the original study on which the present article is based | Submission system |  |

*Give information separately for cases and controls in case-control studies and, if applicable, for exposed and unexposed groups in cohort and cross-sectional studies.

**Note:** An Explanation and Elaboration article discusses each checklist item and gives methodological background and published examples of transparent reporting. The STROBE checklist is best used in conjunction with this article (freely available on the Web sites of PLoS Medicine at http://www.plosmedicine.org/, Annals of Internal Medicine at http://www.annals.org/, and Epidemiology at http://www.epidem.com/). Information on the STROBE Initiative is available at www.strobe-statement.org.
