# Supplementary material for: Characteristics associated with antenatally unidentified small-for-gestational-age fetuses: prospective cohort study nested within the DESiGN randomized control trial
Source: Ultrasound Obstet Gynecol. Author manuscript; Available in PMC 2024 Jun 3. (PMC7616055; doi:10.1002/uog.26091)
Supplement: Appendix 2 [file EMS196131-supplement-Appendix_2.docx]

#### Appendix 2 - Available case sensitivity analysis

***Consort diagram detailing the construction of the study population (complete case data)***


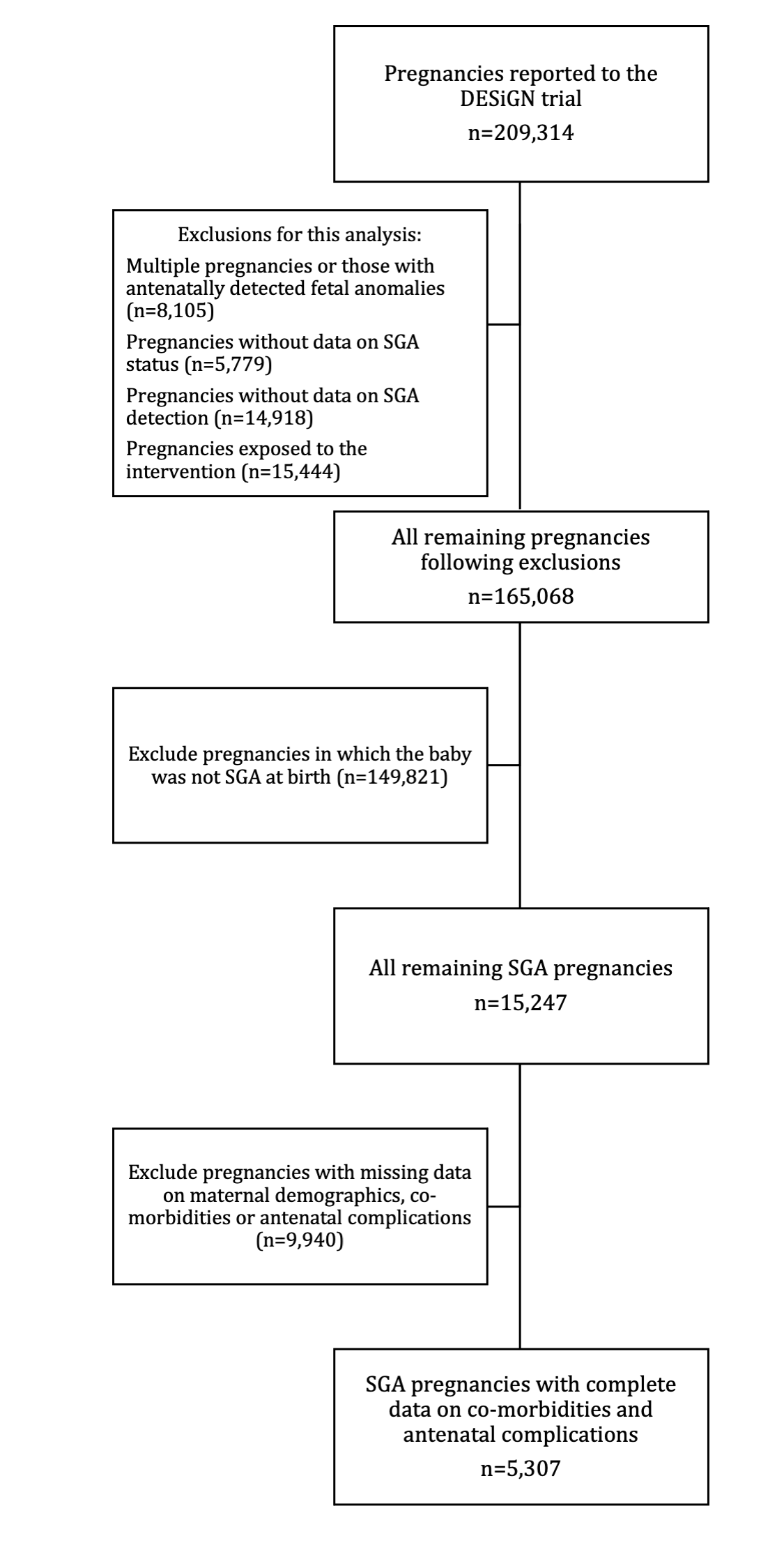


Table A - Characteristics of the included women, by SGA detection status

|  |  | Unidentified SGA (n=4,129, 77.8%) | Identified SGA (n=1,178, 22.2%) | Unadjusted OR (95% CI) | Adjusted OR (95% CI) | Adjusted p value |
| --- | --- | --- | --- | --- | --- | --- |
| Age (years) | **≤40y, n(%)** | 3,961  (78.1%) | 1,110  (21.9%) | Ref | Ref | 0.06 |
|  | **>40y, n(%)** | 168  (71.2%) | 68  (28.8%) | 0.7  (0.5-0.9) | 0.7  (0.5-1.0) |  |
| Index of multiple deprivation (IMD) quintile, n(%) | **1=least deprived** | 386  (73.8%) | 137  (26.2%) | Ref | Ref | 0.25 |
|  | **2** | 436  (74.4%) | 150  (25.6%) | 1.0  (0.8-1.4) | 0.9  (0.7-1.2) |  |
|  | **3** | 1,000  (77.5%) | 291  (22.5%) | 1.2  (0.96-1.5) | 1.1  (0.8-1.4) |  |
|  | **4** | 1,455  (80.1%) | 362  (19.9%) | 1.4  (1.1-1.8) | 1.2  (0.9-1.5) |  |
|  | **5=most deprived** | 852  (78.2%) | 238  (21.8%) | 1.3  (1.0-1.6) | 1.0  (0.8-1.4) |  |
| Ethnicity, n(%) | **White** | 1,663  (78.2%) | 463  (21.8%) | Ref | Ref | 0.09 |
|  | **Black** | 757  (78.3%) | 210  (21.7%) | 1.0  (0.8-1.2) | 0.9  (0.8-1.2) |  |
|  | **Asian** | 1,238  (76.3%) | 385  (23.7%) | 0.9  (0.8-1.0) | 0.8  (0.6-0.9) |  |
|  | **Mixed** | 73  (73.0%) | 27  (27.0%) | 0.8  (0.5-1.2) | 0.9  (0.5-1.4) |  |
|  | **Other** | 398  (81.1%) | 93  (18.9%) | 1.2  (0.9-1.5) | 0.8  (0.6-1.1) |  |
| BMI (kg/m^2^) | **<18.5, n(%)** | 176  (68.2%) | 82  (31.8%) | 0.6  (0.4-0.8) | 0.6  (0.4-0.8) | <0.01 |
|  | **18.5-24.9, n(%)** | 2,221  (78.4%) | 611  (21.6%) | Ref | Ref |  |
|  | **25.0-29.9, n(%)** | 1,121  (79.1%) | 296  (20.9%) | 1.0  (0.9-1.2) | 1.1  (0.9-1.3) |  |
|  | **30.0-34.9, n(%)** | 408  (76.7%) | 124  (23.3%) | 0.9  (0.7-1.1) | 1.0  (0.8-1.3) |  |
|  | **35.0-39.9, n(%)** | 141  (77.9%) | 40  (22.1%) | 1.0  (0.7-1.4) | 1.1  (0.7-1.6) |  |
|  | **≥40.0, n(%)** | 62  (71.3%) | 25  (28.7%) | 0.7  (0.4-1.1) | 0.8  (0.5-1.4) |  |
| Parity, n(%) | **0** | 2,280  (78.5%) | 623  (21.5%) | Ref | Ref | 0.16 |
|  | **1** | 1,159  (76.3%) | 361  (23.8%) | 0.9  (0.8-1.0) | 0.8  (0.7-0.98) |  |
|  | **2** | 408  (78.3%) | 113  (21.7%) | 1.0  (0.8-1.2) | 1.0  (0.7-1.2) |  |
|  | **3** | 165  (75.7%) | 53  (24.3%) | 0.9  (0.6-1.2) | 0.8  (0.6-1.1) |  |
|  | **4 or above** | 117  (80.7%) | 28  (19.3%) | 1.1  (0.7-1.7) | 1.1  (0.7-1.8) |  |
| Smoking, n(%) | **Non-smoker** | 3,784  (78.3%) | 1,047  (21.7%) | Ref | Ref | 0.01 |
|  | **Smoker** | 345  (72.5%) | 131  (27.5%) | 0.7  (0.6-0.9) | 0.7  (0.6-0.9) |  |

Table B - Co-morbidities and fetal characteristics of the included women and babies, by SGA detection status

|  |  | Unidentified SGA (n=4,129) | Identified SGA (n=1,178 | Unadjusted OR (95% CI) | Adjusted OR (95% CI) | Adjusted p value |
| --- | --- | --- | --- | --- | --- | --- |
| Co-morbidities, n(%) | **No hypertension** | 4,063  (78.0%) | 1,143  (22.0%) | Ref | Ref | 0.10 |
|  | **Hypertension** | 66  (65.3%) | 35  (34.7%) | 0.5  (0.4-0.8) | 0.7  (0.4-1.1) |  |
|  | **No diabetes** | 4,074  (77.9%) | 1,159  (22.1%) | Ref | Ref | 0.64 |
|  | **Diabetes** | 55  (74.3%) | 19  (25.7%) | 0.8  (0.5-1.4) | 0.9  (0.5-1.5) |  |
| Antenatal complications, n(%) | **No pre-eclampsia** | 4,040  (78.2%) | 1,124  (21.8%) | Ref | Ref | <0.01 |
|  | **Pre-eclampsia** | 89  (62.2%) | 54  (37.8%) | 0.5  (0.3-0.6) | 0.6  (0.4-0.8) |  |
|  | **No gestational hypertension** | 4,066  (78.0%) | 1,145  (22.0%) | Ref | Ref | 0.04 |
|  | **Gestational hypertension** | 63  (65.6%) | 33  (34.4%) | 0.5  (0.4-0.8) | 0.6  (0.4-0.97) |  |
|  | **No GDM** | 3,940  (78.4%) | 1,086  (21.6%) | Ref | Ref | <0.01 |
|  | **GDM** | 189  (67.3%) | 92  (32.7%) | 0.6  (0.4-0.7) | 0.6  (0.4-0.8) |  |
| PAPP-A, n(%) | **<0.300MoM** | 72  (61.5%) | 45  (38.5%) | 0.5  (0.3-0.7) | 0.7  (0.4-1.1) | 0.10 |
|  | **0.3-0.415MoM** | 130  (62.8%) | 77  (37.2%) | 0.5  (0.4-0.7) | 0.7  (0.5-0.98) | 0.04 |
|  | **>0.415MoM** | 2,003  (76.9%) | 601  (23.1%) | Ref | Ref | Ref |
| Indication for serial fetal scans,† n(%) | **No indication** | 2,645  (81.5%) | 601  (18.5%) | Ref | Ref | <0.01 |
|  | **Any indication** | 1,064  (70.1%) | 454  (29.9%) | 0.5  (0.5-0.6) | 0.6  (0.5-0.7) |  |
| Neonatal presentation at birth, n(%) | **Cephalic** | 2,778  (78.4%) | 767  (21.6%) | Ref | Ref | <0.01 |
|  | **Non-cephalic** | 124  (64.2%) | 69  (35.8%) | 0.5  (0.4-0.7) | 0.5  (0.4-0.7) |  |
| Birthweight centile‡ | **Mean (SD)** | 5.6 (2.8) | 4.1 (2.8) | 1.21  (1.18-1.24) | 1.22  (1.19-1.25) | <0.01 |
| *Information on PAPP-A may be missing. †*Adjusted only for IMD, parity, ethnicity, and allocated birthweight centile (not for other adjustment characteristics which are included in this composite).* ‡*Change in OR with a one centile increase (<10^th^ centile).* | | | | | | |

Table C - Patterns of ultrasound utilisation for all SGA pregnancies, and stratified by presence or absence of a recorded indication for serial fetal growth scans

|  | | All SGA  (n=14,768) | | SGA with serial scan indication | | SGA with no recorded serial scan indication* | |
| --- | --- | --- | --- | --- | --- | --- | --- |
|  | | **Unidentified SGA (n=11,529)** | **Identified SGA (n=3,239)** | **Unidentified SGA (n=1,064)** | **Identified SGA (n=454)** | **Unidentified SGA (n=2,645)** | **Identified SGA (n=601)** |
| Number of screening scans received, % | **0** | 5,184 (45.0%) | - | 296 (27.8%) | - | 1,321 (49.9%) | - |
|  | **1** | 2,575 (22.3%) | 1,809 (55.9%) | 210 (19.7%) | 236 (52.0%) | 583 (22.0%) | 339 (56.4%) |
|  | **2** | 1,769 (15.3%) | 861 (26.6%) | 243 (22.8%) | 121 (26.7%) | 357 (13.5%) | 166 (27.6%) |
|  | **3** | 1,293 (11.2%) | 404 (12.5%) | 211 (19.8%) | 65 (14.3%) | 253 (9.6%) | 65 (10.8%) |
|  | **4** | 497 (4.3%) | 138 (4.3%) | 71 (6.7%) | 24 (5.3%) | 99 (3.7%) | 27 (4.5%) |
|  | **≥5** | 211 (1.8%) | 27 (0.8%) | 33 (3.1%) | 8(1.7%) | 32 (1.2%) | 4 (0.7%) |
| Screening scan frequency for pregnancies with at least two scans: | **≤3-weekly** | 542 (14.4%) | 610 (42.7%) | 84 (15.1%) | 85 (39.0%) | 117 (15.8%) | 110 (42.0%) |
|  | **4-weekly** | 527 (14.0%) | 429 (30.0%) | 70 (12.5%) | 61 (28.0%) | 103 (13.9%) | 69 (26.3%) |
|  | **>4-weekly** | 2,701 (71.6%) | 391 (27.3%) | 404 (72.4%) | 72 (33.0%) | 521 (70.3%) | 83 (31.7%) |
| Gestation at the time of the first scan, if scans conducted, % | **<31^+0^** | 2,931 (46.2%) | 1,818 (56.1%) | 455 (59.2%) | 319 (70.3%) | 595 (44.7%) | 303 (50.4%) |
|  | **31^+0^-33^+6^** | 988 (15.6%) | 446 (13.8%) | 118 (15.4%) | 55 (12.1%) | 191 (14.4%) | 95 (15.8%) |
|  | **34^+0^-36^+6^** | 1,746 (27.5%) | 641 (19.8%) | 147 (19.1%) | 61 (13.4%) | 329 (24.8%) | 129 (21.5%) |
|  | **≥37^+0^** | 680 (10.7%) | 334 (10.3%) | 48 (6.3%) | 19 (4.2%) | 212 (16.0%) | 74 (12.3%) |
| **Includes records for which PAPP-A was not documented.* | | | | | | | |

Table D - Comparison of estimated fetal weight at the last ultrasound scan and the birthweight, including their centiles, for SGA babies born at term

|  |  | Unidentified SGA (n=11,897) | Identified SGA (n=3,350) | Unadjusted OR/mean diff (95% CI) | Adjusted OR/mean diff (95% CI) | p value |
| --- | --- | --- | --- | --- | --- | --- |
| If scan within 1 week*: | | | | | | |
| EFW centile at last scan, mean (SD) |  | 25.5 (13.9) | 4.6 (2.9) | 20.9  (19.8-22.0) | 20.6  (19.5-21.7) | <0.01 |
| Difference between EFW and birthweight centiles, mean (SD) |  | 19.5 (13.6) | 0.2 (3.3) | 19.3  (18.2-20.4) | 19.0  (17.8-20.1) | <0.01 |
| Percentage difference between EFW and birthweight, mean (SD) |  | 13.4% (7.2) | 2.4% (10.9) | 11.0%  (10.1-12.0) | 9.8%  (9.0-10.6) | <0.01 |
| If scan within 2 weeks*: | | | | | | |
| EFW centile at last scan, mean (SD) |  | 26.7 (14.0) | 5.3 (2.8) | 21.5  (19.8-23.1) | 21.2  (19.5-22.8) | <0.01 |
| Difference between EFW and birthweight centiles, mean (SD) |  | 20.9 (13.9) | 0.6 (3.4) | 20.3  (18.7-21.9) | 20.0  (18.4-21.7) | <0.01 |
| Percentage difference between EFW and birthweight, mean (SD) |  | 10.9% (38.0) | -2.6% (9.1) | 13.5%  (9.1-17.9) | 12.9%  (8.5-17.3) | <0.01 |
| If scan within 3 weeks*: | | | | | | |
| EFW centile at last scan, mean (SD) |  | 27.1 (14.1) | 5.4 (2.8) | 21.7  (19.9-23.5) | 21.5  (19.6-23.3) | <0.01 |
| Difference between EFW and birthweight centiles, mean (SD) |  | 21.0 (14.0) | 1.2 (3.6) | 19.8  (18.0-21.6) | 19.7  (17.8-21.5) | <0.01 |
| Percentage difference between EFW and birthweight, mean (SD) |  | 3.2% (27.2%) | -8.1% (12.2) | 11.2%  (7.7-14.8) | 9.2%  (5.6-12.8) | <0.01 |
| If scan within 4 weeks*: | | | | | | |
| EFW centile at last scan, mean (SD) |  | 2.7 (15.0) | 5.6 (3.3) | 24.1  (21.6-26.6) | 24.0  (21.4-26.6) | <0.01 |
| Difference between EFW and birthweight centiles, mean (SD) |  | 23.9 (14.8) | 1.6 (4.2) | 22.3  (19.9-24.7) | 22.1  (19.5-24.6) | <0.01 |
| Percentage difference between EFW and birthweight, mean (SD) |  | -3.0% (9.2) | -13.2% (27.3) | 10.2%  (7.9-12.6) | 5.6%  (3.4-7.9) | <0.01 |
