# Supplementary material for: Characteristics associated with antenatally unidentified small-for-gestational-age fetuses: prospective cohort study nested within the DESiGN randomized control trial
Source: Ultrasound Obstet Gynecol. Author manuscript; Available in PMC 2024 Jun 3. (PMC7616055; doi:10.1002/uog.26091)
Supplement: Appendix 3 [file EMS196131-supplement-Appendix_3.docx]

#### Appendix 3 - Sensitivity analysis for women with a recorded anomaly scan

Table A - Characteristics of women with evidence of a presumed anomaly scan, by SGA detection status

|  |  | Unidentified SGA (n≈5,733, 77.3%) | Identified SGA (n≈1,680, 22.7%) | Unadjusted OR (95% CI) | Adjusted OR (95% CI) | Adjusted p value |
| --- | --- | --- | --- | --- | --- | --- |
| Age (years) | **≤40y (%)** | 77.6% | 22.4% | Ref | Ref | 0.06 |
|  | **>40y (%)** | 70.0% | 30.0% | 0.7  (0.5-0.9) | 0.8  (0.6-1.0) |  |
| IMD, % | **1=least deprived** | 74.9% | 25.1% | Ref | Ref | 0.26 |
|  | **2** | 75.1% | 24.9% | 1.0  (0.8-1.3) | 0.9  (0.7-1.2) |  |
|  | **3** | 78.6% | 21.4% | 1.2  (1.02-1.5) | 1.2  (0.9-1.4) |  |
|  | **4** | 78.4% | 21.6% | 1.2  (1.01-1.5) | 1.1  (0.9-1.3) |  |
|  | **5=most deprived** | 76.3% | 23.7% | 1.1  (0.9-1.3) | 1.0  (0.8-1.3) |  |
| Ethnicity, % | **White** | 78.3% | 21.7% | Ref | Ref | 0.07 |
|  | **Black** | 76.3% | 23.7% | 0.9  (0.8-1.1) | 0.9  (0.7-1.1) |  |
|  | **Asian** | 76.3% | 23.7% | 0.9  (0.8-1.0) | 0.8  (0.7-0.9) |  |
|  | **Mixed** | 71.9% | 28.1% | 0.7  (0.5-1.1) | 0.7  (0.5-1.2) |  |
|  | **Other** | 79.8% | 20.2% | 1.1  (0.9-1.4) | 0.8  (0.6-1.0) |  |
| BMI (kg/m^2^) | **<18.5 (%)** | 68.0% | 32.0% | 0.6  (0.5-0.8) | 0.6  (0.4-0.7) | <0.01 |
|  | **18.5-24.9 (%)** | 77.8% | 22.2% | Ref | Ref |  |
|  | **25.0-29.9 (%)** | 78.9% | 21.1% | 1.1  (0.9-1.2) | 1.2  (1.0-1.4) |  |
|  | **30.0-34.9 (%)** | 77.0% | 23.0% | 1.0  (0.8-1.2) | 1.1  (0.9-1.4) |  |
|  | **35.0-39.9 (%)** | 75.7% | 24.3% | 0.9  (0.7-1.2) | 1.1  (0.8-1.5) |  |
|  | **≥40.0 (%)** | 72.8% | 27.2% | 0.8  (0.5-1.2) | 1.0  (0.6-1.6) |  |
| Parity, % | **0** | 78.2% | 21.8% | Ref | Ref | 0.02 |
|  | **1** | 76.1% | 23.9% | 0.9  (0.8-1.0) | 0.8  (0.7-0.9) |  |
|  | **2** | 76.7% | 23.3% | 0.9  (0.8-1.1) | 0.9  (0.7-1.1) |  |
|  | **3** | 72.9% | 27.1% | 0.8  (0.6-1.0) | 0.7  (0.5-1.0) |  |
|  | **4 or above** | 79.5% | 20.5% | 1.1  (0.7-1.6) | 1.1  (0.7-1.7) |  |
| Smoking, % | **Non-smoker** | 77.8% | 22.2% | Ref | Ref | <0.01 |
|  | **Smoker** | 72.8% | 27.2% | 0.8  (0.6-0.9) | 0.7  (0.6-0.9) |  |

Table B - Co-morbidities and fetal characteristics of the women and babies for which there was evidence of a presumed anomaly scan, by SGA detection status

|  |  | Unidentified SGA (n≈5,733) | Identified SGA (n≈1,680) | Unadjusted OR (95% CI) | Adjusted OR (95% CI) | Adjusted p value |
| --- | --- | --- | --- | --- | --- | --- |
| Co-morbidities, % | **No hypertension** | 77.6% | 22.4% | Ref | Ref | <0.01 |
|  | **Hypertension** | 64.0% | 36.0% | 0.5  (0.4-0.7) | 0.6  (0.4-0.9) |  |
|  | **No diabetes** | 77.5% | 22.5% | Ref | Ref | <0.01 |
|  | **Diabetes** | 63.7% | 36.3% | 0.5  (0.3-0.8) | 0.5  (0.3-0.8) |  |
| Antenatal complications, % | **No pre-eclampsia** | 78.1% | 21.9% | Ref | Ref | <0.01 |
|  | **Pre-eclampsia** | 55.4% | 44.6% | 0.4  (0.3-0.5) | 0.4  (0.3-0.5) |  |
|  | **No PIH** | 77.6% | 22.4% | Ref | Ref | <0.01 |
|  | **PIH** | 64.1% | 35.9% | 0.5  (0.4-0.7) | 0.6  (0.4-0.8) |  |
|  | **No GDM** | 77.8% | 22.2% | Ref | Ref | <0.01 |
|  | **GDM** | 68.8% | 31.2% | 0.6  (0.5-0.8) | 0.6  (0.5-0.7) |  |
| PAPP-A, % | **<0.300MoM** | 57.7% | 42.3% | 0.4  (0.3-0.6) | 0.4  (0.3-0.6) | <0.01 |
|  | **0.3-0.415MoM** | 63.4% | 36.6% | 0.5  (0.4-1.3) | 0.6  (0.4-0.7) |  |
|  | **>0.415MoM** | 77.5% | 22.5% | Ref | Ref |  |
| Indication for serial fetal scans,† % | **No indication** | 80.9% | 19.1% | Ref | Ref | <0.01 |
|  | **Any indication** | 68.4% | 31.6% | 0.5  (0.5-0.6) | 0.5  (0.5-0.6) |  |
| Neonatal presentation at birth, % | **Cephalic** | 78.6% | 21.4% | Ref | Ref | <0.01 |
|  | **Non-cephalic** | 63.5% | 36.5% | 0.5  (0.4-0.6) | 0.5  (0.4-0.7) |  |
| Birthweight centile‡ | **Mean (SD)** | 5.6 (2.8) | 4.0 (2.8) | 1.2  (1.2-1.3) | 1.2  (1.2-1..3) | <0.01 |
| *Complete case data except that information on PAPP-A may be missing. †*Adjusted only for IMD, parity, ethnicity, and allocated birthweight centile (not for other adjustment characteristics which are included in this composite).* ‡*Change in OR with a one centile increase (<10^th^ centile).* | | | | | | |

Table C - Patterns of ultrasound use for SGA pregnancies with evidence of a presumed anomaly scan, and stratified by presence or absence of a recorded indication for serial fetal growth scans

|  | | All SGA  (n≈12,122) | | SGA with serial scan indication | | SGA with no recorded serial scan indication* | |
| --- | --- | --- | --- | --- | --- | --- | --- |
|  | | **Unidentified SGA (n≈9,164)** | **Identified SGA (n≈2,958)** | **Unidentified SGA (n≈1,230)** | **Identified SGA (n≈568)** | **Missed SGA (n≈3,176)** | **Detected SGA (n≈750)** |
| Number of screening scans received, % | **0** | 36.6% | - | 23.4% | - | 49.0% | - |
|  | **1** | 25.4% | 55.2% | 21.3% | 52.7% | 23.3% | 58.2% |
|  | **2** | 17.5% | 27.0% | 24.0% | 26.4% | 13.8% | 26.4% |
|  | **3** | 13.3% | 12.7% | 21.5% | 14.9% | 9.4% | 10.4% |
|  | **4** | 5.0% | 4.3% | 6.6% | 4.6% | 3.2% | 4.5% |
|  | **≥5** | 2.3% | 0.9% | 3.3% | 1.5% | 1.2% | 0.5% |
| Screening scan frequency for pregnancies with at least two scans: | **≤3-weekly** | 14.5% | 42.4% | 14.6% | 42.6% | 15.1% | 42.9% |
|  | **4-weekly** | 14.1% | 30.5% | 12.3% | 26.5% | 13.5% | 25.8% |
|  | **>4-weekly** | 71.5% | 27.1% | 73.1% | 30.9% | 71.3% | 31.3% |
| Gestation at the time of the first scan, if scans conducted, % | **<31^+0^** | 47.0% | 57.2% | 59.9% | 70.9% | 42.3% | 47.9% |
|  | **31^+0^-33^+6^** | 15.0% | 13.4% | 14.5% | 11.3% | 15.4% | 15.6% |
|  | **34^+0^-36^+6^** | 27.8% | 19.8% | 20.2% | 13.6% | 26.5% | 22.5% |
|  | **≥37^+0^** | 10.2% | 9.6% | 5.3% | 4.1% | 15.8% | 14.0% |
| **Includes records for which PAPP-A was not documented.* | | | | | | | |

Table D - Comparison of estimated fetal weight at the last ultrasound scan and the birthweight, including their centiles, for SGA babies born at term, from pregnancies with evidence of a presumed anomaly scan.

|  |  | Unidentified SGA (n≈9,502) | Identified SGA (n≈3,055) | Unadjusted OR/mean diff (95% CI) | Adjusted OR/mean diff (95% CI) | p value |
| --- | --- | --- | --- | --- | --- | --- |
| If scan within 1 week*: | | | | | | |
| EFW centile at last scan, mean (SD) |  | 25.4 (13.8) | 4.6 (2.8) | 20.8  (19.6-21.9) | 20.4  (19.2-21.6) | <0.01 |
| Difference between EFW and birthweight centiles, mean (SD) |  | 19.3(13.5) | 0.2 (3.3) | 19.1  (17.9-20.2) | 18.7  (17.6-19.9) | <0.01 |
| Percentage difference between EFW and birthweight, mean (SD) |  | 13.3% (7.2) | 2.3% (11.2) | 11.0%  (10.0-12.0) | 9.6%  (8.8-10.4) | <0.01 |
| If scan within 2 weeks*: | | | | | | |
| EFW centile at last scan, mean (SD) |  | 26.5 (13.8) | 5.4 (2.8) | 21.1  (19.4-22.8) | 20.9  (19.2-22.6) | <0.01 |
| Difference between EFW and birthweight centiles, mean (SD) |  | 20.7 (13.6) | 0.8 (3.4) | 19.9  (18.2-21.6) | 19.8  (18.1-21.5) | <0.01 |
| Percentage difference between EFW and birthweight, mean (SD) |  | 11.1% (40.2) | -2.0% (8.4) | 13.1%  (8.1-18.0) | 12.8%  (7.8-17.8) | <0.01 |
| If scan within 3 weeks*: | | | | | | |
| EFW centile at last scan, mean (SD) |  | 27.1 (13.8) | 5.3 (2.7) | 21.7  (19.9-23.5) | 21.5  (19.7-23.4) | <0.01 |
| Difference between EFW and birthweight centiles, mean (SD) |  | 21.0 (13.7) | 1.2 (3.6) | 19.8  (18.0-21.6) | 19.7  (17.8-21.5) | <0.01 |
| Percentage difference between EFW and birthweight, mean (SD) |  | 3.3% (28.2) | -7.7% (11.6) | 11.0%  (7.2-14.8) | 9.2%  (5.4-13.1%) | <0.01 |
| If scan within 4 weeks*: | | | | | | |
| EFW centile at last scan, mean (SD) |  | 30.0 (15.2) | 5.6 (3.3) | 24.3  (21.7-26.9) | 24.3  (21.5-27.1) | <0.01 |
| Difference between EFW and birthweight centiles, mean (SD) |  | 24.2 (15.1) | 1.7 (4.3) | 22.5  (19.9-25.1) | 22.3  (19.6-25.1) | <0.01 |
| Percentage difference between EFW and birthweight, mean (SD) |  | -2.9% (9.4%) | -12.7% (28.1%) | 9.8%  (7.3-12.3) | 5.5%  (3.1-7.9) | <0.01 |
